# Deficits in spatial learning and motor coordination in ADAM11-deficient mice

**DOI:** 10.1186/1471-2202-7-19

**Published:** 2006-02-26

**Authors:** Eiki Takahashi, Koji Sagane, Tohru Oki, Kazuto Yamazaki, Takeshi Nagasu, Junro Kuromitsu

**Affiliations:** 1 Tsukuba Research Laboratories, Eisai Co., Ltd., 5-1-3 Tokodai, Tsukuba, Ibaraki 300-2635, Japan

## Abstract

**Background:**

ADAM11 is a member of the ADAM gene family and is mainly expressed in the nervous system. It is thought to be an adhesion molecule, since it has a disintegrin-like domain related to cell-cell or cell-matrix interactions. To elucidate the physiological functions of ADAM11, we generated ADAM11-deficient mice by means of gene targeting.

**Results:**

ADAM11-deficient mice were apparently normal, and survived more than one year with no major histological abnormalities in the brain or spinal cord. Because ADAM11 is highly expressed in the hippocampus and cerebellum, we have examined ADAM11 mutant mice for learning using visual and hidden water maze tasks, and their motor coordination using a rotating rod task. Our results showed that their visual water maze task results are normal, but the hidden water maze and rotating rod task skills are impaired in ADAM11-deficient mice.

**Conclusion:**

Our results indicate that ADAM11 mutation does not affect cell migration and differentiation during development, but affects learning and motor coordination. Thus, ADAM11 might play an important signalling or structural role as a cell adhesion molecule at the synapse, and may thus participate in synaptic regulation underlying behavioural changes.

## Background

The ADAM (A Disintegrin And Metalloprotease) family comprises membrane-spanning multi-domain proteins containing a metalloproteinase-like domain and a disintegrin-like domain. Approximately half of the ADAMs are catalytically active metalloproteases that shed a broad range of substrates, such as cytokines, growth factors, receptors, adhesion molecules [1,2]. Recent gene-disruption studies in mice have disclosed a physiological role of each ADAM. For example, ADAM10, ADAM17 and ADAM19 are critical for normal development by providing growth signals at the correct times and places [3-6]. In contrast, the functions of ADAM8, ADAM9, ADAM12 and ADAM15 are not essential for embryogenesis, suggesting a possible functional redundancy with other protease [7-10]. The remaining half, which are non-protease-ADAMs, are thought to be adhesion molecules. More than ten ADAMs have been shown to support integrin-mediated cell adhesion *in vitro *[11]. More recently, the finding has been reported that integrin-mediated cell migration of tissue culture cells can be controlled by distinct ADAMs [12]. In *C. elegans*, *unc-71 *gene coding a non-protease-ADAM protein have been shown to be involved in cell migration events *in vivo *[13]. However, the physiological roles of mammalian non-protease-ADAMs are poorly understood *in vivo*. Genetic deletion studies in mice have shown that both *Adam2*-null and *Adam3*-null male mutants are infertile and their spermatozoa lack egg-binding abilities [14-16]. The precise mechanism is under investigation [17].

We have reported the findings of ADAM11, ADAM22 and ADAM23 genes and their restricted expression in the human and murine nervous systems [18,19]. These ADAMs lack a catalytic motif critical for the metalloproteinase activity, suggesting that they are not proteases. We assume that these ADAMs might contribute to cell migration, differentiation and survival or synaptic regulation by binding to integrins, cell-surface proteins or intracellular signalling molecules [20,21]. We have reported severe ataxia, seizures, and death before weaning, accompanied with hypomyelination of the peripheral nervous system in *Adam22*-null mutant mice [22]. It has been reported that the disruption of *Adam23 *gene in the mouse results in premature death associated with ataxia and tremor [23]. Is spite of such extreme phenotypes, no histological defects in the brain has been observed in either *Adam22*-null nor *Adam23*-null mice.

ADAM11 is the closest paralog of ADAM22 and ADAM23, and its orthologs have been found in vertebrates, but not in invertebrates. It has been reported that the *Xenopus *ADAM11 ortholog, xMDC11a, has an expression pattern associated with neural development, with a proposed role in cell migration [24]; and it has been reported in analyses using Northern blot and *in situ *hybridisation methods that the murine *Adam11 *gene is expressed in both the developing and adult nervous system [19,25]. These findings led us to hypothesise that ADAM11 is an integrin binder that plays an important role in the nervous system, as do ADAM22 and ADAM23. To determine the physiological functions of ADAM11, we generated and analysed *Adam11 *gene-targeted mice.

## Results

### Generation of ADAM11-deficient mice

**Figure 1:**
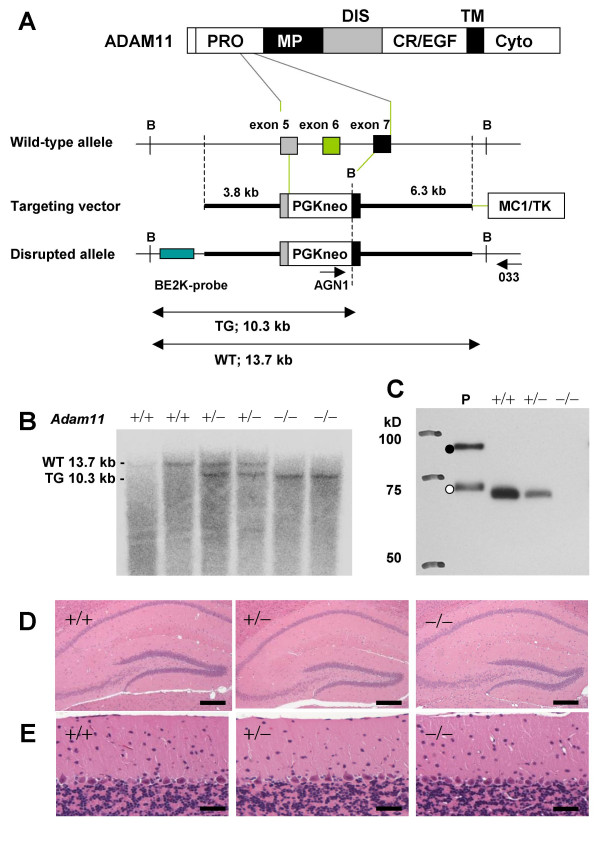
**Targeted mutation of *Adam11***. (A) Top; Domain structure of ADAM11 protein. PRO, proprotein; MP, metalloprotease-like; DIS, disintegrin-like; CR/EGF, Cysteine-rich and EGF-like repeat; TM, transmembrane; Cyto, cytoplasmic domain. *Adam11 *gene was disrupted by the insertion of a termination codon and a PGKneo cassette into exons 5 – 7. An MC1/TK cassette at the end of the targeting vector allowed for negative selection. The BE2K-probe used for Southern blot analysis, and the expected *Bam*HI "B" fragments of the targeted allele (TG) and wild-type allele (WT) are indicated by arrows. The primers, AGN1 and 033 were used for the selection of the recombinant ES clones. (B) Southern blot analysis of mouse genomic DNA. The expected DNA fragments for the wild-type allele (WT) and targeted allele (TG) are 13.7-kb and 10.3-kb, respectively. +/+, wild-type; +/-, heterozygote; -/-, homozygote. (C) Western blot analysis of ADAM11 expression in the mouse cerebellum. Absence of ADAM11 protein in the (-/-) mutant cerebellum was shown using anti-ADAM11 monoclonal antibody. "P" indicates the positive control, HA-tagged mouse ADAM11 protein expressed in HeLa cells. White circle indicates mature-form of ADAM11 protein and black circle shows immature-form, which was not processed. (D) Hippocampus sections with HE stain. Scar = 30 μm. (E) Cerebellum sections with HE stain. Scar = 200 μm.

Mice carrying a targeted mutation in their *Adam11 *gene were generated by homologous recombination (Fig. 1A). Correct targeting events were confirmed by Southern blot analysis (Fig. 1B). As a result of this procedure, since a termination codon was introduced in exon 5 in the pro-protein domain, only the truncated form of the ADAM11 protein would be synthesised from this targeted allele. Because pro-protein domains are always removed in mature functional ADAM-proteins by furin-like proteases and are thus thought to be non-functional, we concluded that this truncated form of ADAM11 protein would have no function [26]. Absence of mature ADAM11 protein in homozygous mutants was confirmed by Western blot analysis using a specific antibody that recognises the ADAM11 protein (Fig. 1C). Analysis by Western blot showed that heterozygous mutants produced approximately half of the wild-type levels of ADAM11 protein.

### ADAM11-deficient mice are viable and appear normal

Mating of mice heterozygous for the ADAM11 mutation yielded a near-Mendelian distribution of genotypes in the offspring and the ratio of females and males was the same in all groups (data not shown). For the present experiments, ADAM11-deficient male mice (backcrossed generations into C57BL/6NCrj, n = 14) were used in all behavioural analyses and anatomical-histological studies. Homozygous ADAM11-deficient mice were viable and appeared normal, indicating that the *Adam11 *gene is not essential to development and survival. The gain in body weight of (-/-) mice was comparable to that of (+/+) and (+/-) mice at least until 24 weeks of age (data not shown). There was no significant difference in locomotor activity among (+/+), (+/-) and (-/-) genotypes (data not shown). No apparent anatomical-histological change was observed in the spinal cord, heart, lung, liver, kidney, spleen, testis or ovary (data not shown). The brain of (-/-) mice, including the hippocampus (Fig. 1D) and cerebellum (Fig. 1E) appeared normal after HE staining when (+/+), (+/-) and (-/-) mice were compared. Since *Adam11 *is predominantly expressed in the hippocampus and cerebellum, we applied hippocampus- or cerebellum-dependent behavioural analyses.

### Spatial learning in ADAM11-deficient mice

**Figure 2:**
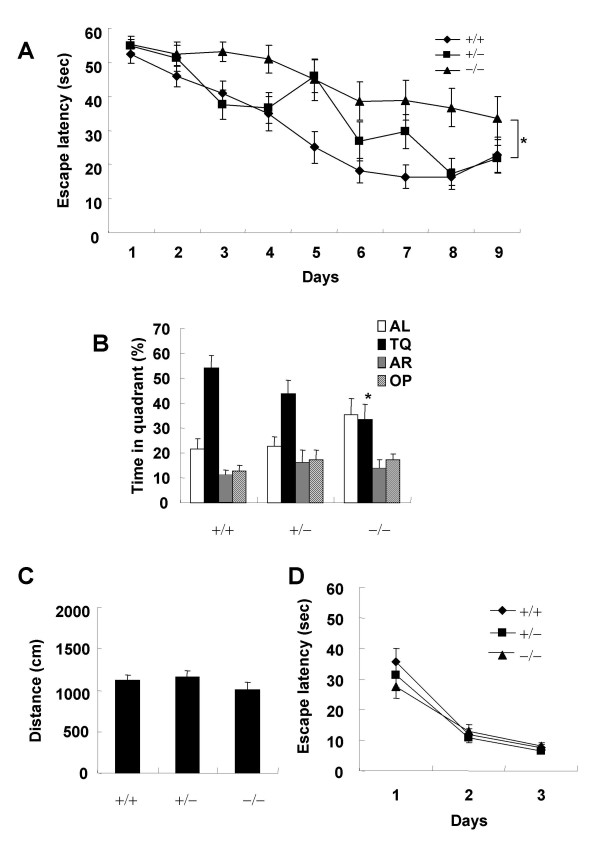
**Morris water maze task of ADAM11-deficient mice**. (A) Escape latencies (sec) in the hidden platform task over nine testing days. Asterisk: significant (p < 0.05) difference from (+/+) mice. (B) The probe test after the acquisition trials of the hidden platform task. Times spent in each quadrant of the water pool are shown. Asterisk: significant (p < 0.05) difference from (+/+) mice. TQ, training quadrant; AR, adjacent right quadrant; AL, adjacent left quadrant; OP, opposite quadrant. (C) Swimming distances (cm) during the probe test. (D) Escape latencies (sec) in the visible platform task over three testing days. +/+, +/- and -/-; n = 11, 14 and 12, respectively.

To study the functions of ADAM11 in the hippocampus, we examined the spatial learning ability of ADAM11-deficient mice using the Morris Water Maze task [27]. First, mice were trained in a hidden platform task, in which mice searched for a submerged platform to escape from the water. *Adam11 *(-/-) mice were found to take a significantly longer time to locate the hidden platform than (+/+) and (+/-) mice (Fig. 2A). Nonetheless, the ability of (+/+), (+/-) and (-/-) mice to find the hidden platform improved over test sessions (Fig. 2A). Thus, (+/+), (+/-) and (-/-) mice were able to learn the location of the hidden platform during the course of the trials, although the capacity of (-/-) mice to find the platform was lower than that of (+/+) and (+/-) mice. We further performed a probe test, in which the platform was removed from the pool after completion of the hidden platform task, and the trained mice were allowed to swim freely for 60 sec. The time spent in the target quadrant of (-/-) is significantly lower than that of (+/+) mice (Fig. 2B). *Adam11 *(+/-) mice appeared to spend a shorter time in the target quadrant, but there was no significant difference between (+/+) and (+/-) mice (Fig. 2B). These results indicate a spatial learning impairment in (-/-) mice. The swimming distance of (+/+), (+/-) and (-/-) mice, however, was similar during the probe trial (Fig. 2C). In the visible platform task, the performance of mice with (+/+), (+/-) and (-/-) genotypes improved during the course of the test sessions, as indicated by a progressive decrease in the latency for locating the platform (Fig. 2D), suggesting that (-/-) mice learn this visible platform task just as well as (+/+) and (+/-) mice. These results also indicated that (-/-) mice have normal visible and motor function and the motivation needed in the water maze task.

### Motor function of ADAM11-deficient mice

**Figure 3:**
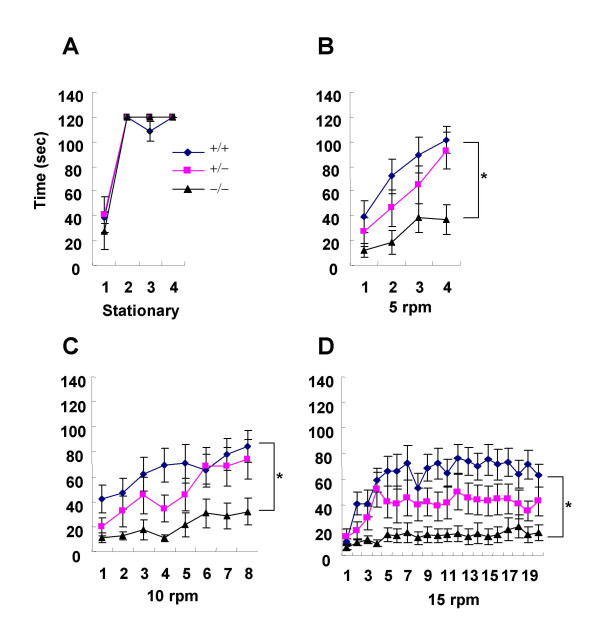
**Retention time of ADAM11-deficient mice on the rotating rod at speeds of 0 rpm (stationary), 5, 10 and 15 rpm**. Asterisk: significant (p < 0.05) difference from (+/+) mice. +/+, +/- and -/-; n = 11, 14 and 12, respectively.

**Figure 4:**
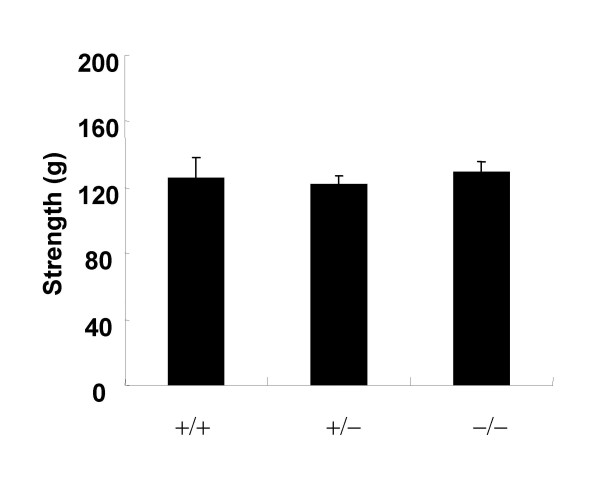
**Traction test (g) of ADAM11-deficient mice**. +/+, +/- and -/-; n = 12, 10 and 12, respectively.

**Figure 5:**
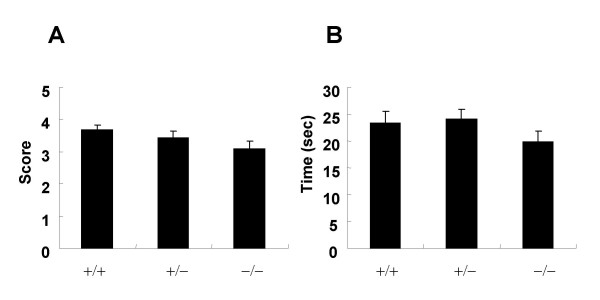
**Wire suspension test of ADAM11-deficient mice**. (A) Score in the wire suspension test. (B) Retention time (sec) in the wire suspension test. +/+, +/- and -/-; n = 12, 10 and 12, respectively.

**Figure 6:**
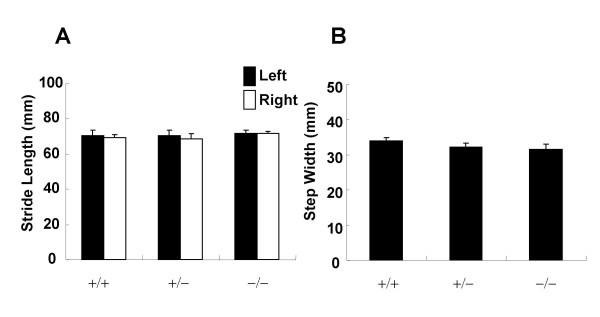
**Footprint test of ADAM11-deficient mice**. (A) Stride length (cm) in the footprint test. (B) Step width (cm) in the footprint test. +/+, +/- and -/-; n = 12, 10 and 12, respectively.

To study the functions of ADAM11 in the cerebellum, we examined the motor function of ADAM11-deficient mice using a rotating rod task [28]. In the rotating rod test, the retention time of (+/-) and (-/-) mice on the rod was not significantly different from that of (+/+) mice at 0 rpm (stationary) [+/-: *F *(1, 80) = 1.67, *p *= 0.21; -/-: *F *(1, 88) = 0.002, *p *= 0.96] (Fig. 3A). In (+/-) mice, the tendency of decreased retention times was seen at 5, 10 and 15 rpm, but there was no significant difference between (+/+) and (+/-) mice [5 rpm: *F *(1, 20) = 1.27, *p *= 0.27; 10 rpm: *F *(1, 20) = 1.07, *p *= 0.31; 15 rpm: *F *(1, 20) = 2.716, *p *= 0.11] (Figs. 3B, 3C and 3D). However, (-/-) mice fell more quickly from the rod than (+/+) mice at 5, 10 and 15 rpm [5 rpm: *F *(1, 27.3) = 17.51, *p *= 0.0002; 10 rpm: *F *(1, 22) = 11.05, *p *= 0.003; 15 rpm: *F *(1, 22) = 19.22, *p *= 0.0002] (Figs. 3B, 3C and 3D). The retention time was not significantly improved in (-/-) mice across trials of training at 5, 10 and 15 rpm [5 rpm: *F *(3, 15.3) = 2.05, *p *= 0.15; 10 rpm: *F *(7, 11) = 2.61, *p *= 0.07; 15 rpm: *F *(19, 26.6) = 1.57, *p *= 0.14] (Figs. 3B, 3C and 3D). In a traction test, (-/-) mice showed a high grip strength similar to that of (+/+) and (+/-) mice (Fig. 4). The hanging score and time of (-/-) mice were also similar to those of (+/+) and (+/-) mice (Figs. 5A and 5B). A footprint test did not reveal any abnormalities in walking patterns, including stride length and step width, as a function of genotypes (Figs. 6A and 6B).

## Discussion

In the present study, we examined the function of ADAM11 by generating mice that lacked the *Adam11 *gene. It has been reported that the *Adam11 *gene is expressed in the brain, heart, liver and testis, as manifested in Northern blot analysis [19]. Interestingly, ADAM11-deficient mice appeared normal and survived more than one year without apparent histological abnormality, including the brain, heart, liver and testis. The expression patterns of the *Adam11 *gene in the brain have been studied using *in situ *hybridisation [25]. This study has shown high expression of ADAM11 mRNA in the hippocampus and cerebellum. We therefore focused on examining the hippocampus- and cerebellum-dependent behavioural tests to study the functions of ADAM11 in the nervous system.

It is widely accepted that the hippocampus is involved in the performance of the hidden platform version of the water maze test, since lesions of the hippocampal pathways lead to a severe impairment of learning in this task [29]. In the hidden platform task, ADAM11-deficient mice needed more time to reach the platform than wild-type mice. Spatial learning was also assessed using a probe trial, in which the platform was removed from the pool on the day following nine days of training. The wild-type mice spent significantly more time searching for the platform in the target quadrant than the other quadrants. However, ADAM11-deficient mice did not search selectively in the target quadrant. On the other hand, in the visible platform version, in which the motor ability and visual acuity of the mouse are tested, ADAM11-defieient mice were normal. These results indicated that ADAM11-deficient mice showed a spatial learning impairment.

The cerebellum is in charge of the smooth coordination of somatic motor activity, regulation of muscle tone, and mechanisms that influence and maintain equilibrium [30]. It is widely accepted that the cerebellum is involved in the performance of the rotating rod task, since mice with structural abnormalities in the cerebellum [31] or with disruptions in genes enriched in the cerebellum [32] exhibit performance deficits in this task. In the rotating rod test in this study, although the retention time of ADAM11-deficient mice on the rod was not different from wild-type mice at 0 rpm (stationary), ADAM11-deficient mice fell more quickly from the rod than wild-type mice at 5, 10 and 15 rpm. These results indicate that ADAM11-deficient mice have a deficit in motor coordination. On the other hand, in the visual platform version of the water maze task, motor ability for swimming in ADAM11-deficient mice was normal. These results suggested that there might be a different mechanism of motor function between swimming and the rotating rod task. It has been also reported that the cerebellum is involved in another important distinct function: learning associated with component movement (motor learning) [33]. The retention time of synaptotagmin (Syt) IV-deficient mice on the rod was significantly shorter than wild-type mice, and they improved it approximately to the same rates as did wild-type mice during rotating rod performance [34]. These data suggest the basis for the performance deficit on the rotating rod task in the Syt IV mutants is in motor coordination rather than in motor learning. The results in the present study showed that ADAM11-deficient mice slightly, though not significantly, improved motor learning ability during rotating rod performance. However, the deficit of motor learning in ADAM11-deficient mice was not clear in this study. Because they could not ride on the rotating rod, they might not learn this task. To examine motor learning ability in ADAM11-deficient mice in detail, we will need to use other cerebellar-dependent learning tasks, such as a classical eyelid conditioning test. There was no impairment in the grip strength and wire suspension test in ADAM11-deficient mice. Spontaneous motor activity and walking patterns of ADAM11-deficient mice were also found to be normal, suggesting that the dysfunction found in the rotating rod test was not derived from muscle weakness or other peripheral disturbances.

These results suggest that limited brain sites, mainly the hippocampus and cerebellum, might contribute to the dominant phenotype, spatial learning impairment and motor discoordination in ADAM11-deficient mice. *In situ *hybridisation analysis has detected *Adam11 *gene expression in the pyramidal cells of CA1–CA3 fields and granule cells of the dentate gyrus in the hippocampus and in granular cells in the cerebellum [25]. It is unlikely that morphological changes during development led to impairment of spatial learning and motor coordination, and morphological alterations in the cytoarchitecture of the hippocampus and cerebellum were not observed in ADAM11-deficient mice after histological investigation using HE staining. However, because it is thought that ADAM11 plays a role in neuron-neuron or neuron-glial cell interactions, a more precise morphological investigation will be needed. It has been reported that adhesion molecules, such as neural cell adhesion molecule (NCAM), are not only implicated in cell interactions during nervous system development, but are also recognised as important mediators of synaptic plasticity in the adult nervous system [35]. Changes in neuron excitability in the hippocampus, such as decreases in the post-burst after hyperpolarization (AHP), have been thought to affect long-term potentiation (LTP) [36], an experimental model of the synaptic changes thought to underlie learning and memory [37]. It has also been reported that mice lacking NMDA receptor for both NR2A and NR2C subunits showed motor dysfunction and complete loss of both spontaneous and evoked excitatory postsynaptic currents (EPSCs) in cerebellar granule cells [38]. Therefore, electrophyological studies to enable analysis of synaptic transmission and plasticity in the hippocampus and cerebellum of ADAM11-deficient mice will be needed. These studies might clarify whether ADAM11 plays an important signalling or structural role at the synaptic level and whether it participates in synaptic regulation.

ADAM11-deficient mice remained alive for more than one year without ataxia or tremor, but showed a deficit in spatial learning and motor dysfunction. On the other hand, mice lacking the *Adam22 *gene showed severe ataxia, exhibited marked hypomyelination of the peripheral nerves, and died before weaning [22]. Mice lacking the *Adam23 *gene showed severe ataxia and tremor and died before weaning [23]. These findings suggest that ADAM11, ADAM22 and ADAM23 have separate and important roles in the nervous system. A genome database search revealed orthologs of ADAM11, ADAM22 and ADAM23 genes to exist in vertebrates such as mammals, fish, and amphibians, but not in invertebrates. Although the precise molecular functions of these ADAMs are still unclear, further investigations will provide clues to understanding the nervous system of higher organisms.

## Conclusion

ADAM11-deficient mice survived more than one year without ataxia or tremor, showed no abnormalities in body weight gain, spontaneous motor activity, muscle strength, or walking patterns, and exhibited no apparent anatomical-histological changes. However, hippocampus-dependent spatial learning and cerebellum-dependent motor coordination in ADAM11-deficient mice were impaired when tested using water maze and rotating rod tasks. Our genetically modified mouse strain has the potential to be a useful and sensitive tools for detailed investigation of ADAM proteins' functions in the nervous system.

## Methods

### Construction of an Adam11 gene-targeting vector

The 13.7-kb *Bam*HI DNA fragment covering exons 2 – 20 of the *Adam11 *gene was amplified from C57BL/6 genomic DNA by the PCR using Expand Hi-Fidelity polymerase mix (Roche Diagnostics KK, Tokyo, Japan). To generate a mutation in the mouse *Adam11 *gene, we inserted a PGKneo cassette into exons 5 – 7 of the *Adam11 *gene. The final targeting construct consisted of a 10.1-kb genomic DNA fragment that was interrupted by the insertion of the PGKneo cassette and contained a MC1/TK cassette as a negative selection marker against random integration [39].

### Generation of Adam11 deficient mice

The linearised targeting vector was electroporated into TT2 embryonic stem (ES) cells [40]. Homologous recombinants were selected by PCR using the following primers, AGN1: 5'-TCGTGCTTTACGGTATCGCCGCTCCCGATT-3' in the PGKneo cassette, and SGN033: 5'-GGACCCGGAAGACATTTGCACGGT-3' outside the targeting vector. Homologous recombined DNA was efficiently amplified by 35 cycles of the following amplification steps (94°C-30 sec and 68°C-5 min). Correctly targeted ES clones were injected into fertilised ICR mouse eggs at the eight-cell stage. The resulting male chimeras were mated with C57BL/6NCrj females (Charles River Japan, Tokyo, Japan), and resulting heterozygous (+/-) male and female mice were interbred to generate homozygous (-/-) mice.

### Southern blot analysis

Mouse genomic DNA used for Southern blot analysis was extracted from the liver of each genotype. *Bam*HI-digested genomic DNA was probed with the [^32^P]-labelled BE2K probe, which was a 2.0-kb *Bam*HI – *Eco*RI genomic fragment located in intron 2.

### Western blot analysis

The absence of ADAM11 protein in the (-/-) mice was confirmed by Western blot analysis. To be brief, the cerebellum was isolated from a six month-old mouse of each genotype and homogenised in cell lysis buffer (50 mM Tris-HCl, pH 7.5, 100 mM NaCl, 1% NP-40, protease inhibitor cocktail [Roche Diagnostics]). After removal of cell debris by centrifugation, the glycosylated proteins in the supernatant were concentrated by the affinity chromatography using Con A Sepharose 4B (Amersham Biosciences Corp.). Each sample was separated on 10% SDS-PAGE, and transferred to a PVDF membrane. The blot was then incubated with monoclonal antibody against the ADAM11 (U. S. Patent 5,631,351) at 1 μg/ml. Bound antibodies were visualised with horseradish peroxidase-labelled second antibody and an ECL-plus chemiluminescence detection system (Amersham Biosciences Corp.).

### Behavioural analysis

All animal procedures conformed to the Japanese regulations on animal care and use, following the Guideline for Animal Experimentation of the Japanese Association for Laboratory of Animal Science, and were approved by the Animal Care and Use Committee of Eisai Co., Ltd. For the present experiments, ADAM11-deficient male mice (backcrossed generations into C57BL/6NCrj, n = 14) were used in all behavioural analyses (22–24 weeks of age). All of the behavioural studies were conducted between 10:00 and 16:00 by a well-trained experimenter blind to the mouse genotypes.

### Spontaneous motor activity

Locomotor activity in a novel environment was measured by introducing mice (+/+, +/- and -/-; n = 8, 8 and 8, respectively) for 90 min in a Plexiglas box (30 × 20 × 13 cm) using a VERSAMAX equipment and software (Accuscan, Columbus, OH, USA).

### Water maze task

Spatial learning was assessed by three variants of the Morris water maze task [27] adapted for mice. *Hidden platform task*: The pool was divided into four quadrants with four starting locations, called north, east, south and west, at equal distances to the rim. A circular, transparent acrylic platform (diameter 8 cm) was submerged 1 cm below the surface of the water in the centre of the southeast quadrant for each trial of this task. Each mouse was allowed to swim for 60 sec and the time required to reach the platform (escape latency) was recorded in each trial. In total this task consisted of four trials per day over 9 days (1 min inter-trial intervals). Each trial was initiated by randomly placing an animal in one of the four starting locations. *Probe trial*: A single probe trial was carried out after the hidden platform task had been completed. In this trial, the platform was removed and the movement of each mouse was monitored using a computer-based video tracking system (BTA-2; Muromachi Kikai Co., Ltd., Tokyo, Japan). Each mouse was placed into the northwest quadrant of the pool and allowed to swim for 60 sec. Swim path length and the time spent in the trained (southeast) quadrant were calculated. *Visible platform task*: In this task, the circular platform was made visible by attaching a black board to it and the mouse was required to locate the visible platform. The mouse was allowed to swim for 60 sec and this task consisted of four trials per day for three consecutive days (1 min inter-trial intervals). Each trial was initiated by randomly placing an animal in one of the four starting locations.

### Rotating rod test

Motor coordination was assessed with a rotating rod apparatus (KN-75, Natsume Seisakujo, Tokyo, Japan), which consisted of a plastic rod (3 cm diameter, 8 cm long) with a gritted surface flanked by two large discs (40 cm diameter). The mice were first placed on the stationary rod for 4 successive trials, followed by 4 trials at a rotation speed of 5 rpm, 8 trials at 10 rpm, and 20 trials at 15 rpm in that order. Latency until a fall occurred was monitored for 120 sec. Intra-trial intervals for each animal were more than 20 min.

### Traction test

The grip strength of each mouse was measured with a traction apparatus (FU-1, Muromachi Kikai Co., Ltd.). Each mouse was made to grasp the attached bar (2 mm diameter) with the forepaws and was slowly pulled back by the tail. The maximum tension before release was recorded.

### Wire suspension test

A mouse was placed on a stainless bar (50 cm length, 2 mm diameter, elevated up to 37 cm from a surface) at a point midway between the supports and observed for 30 sec in four separate trials. The amount of time spent hanging was recorded and scored according to the following system [41]: 0, fell off; 1, hung onto the bar with two forepaws; 2, in addition to 1, attempted to climb onto the bar; 3, hung onto the bar with two forepaws and one or both hind paws; 4, hung onto the bar with all four paws with tail wrapped around the bar; 5, escaped to one of the supports.

### Footprint test

Black ink was applied to the hind paws of each mouse and the mice were placed in a narrow alley (9 × 25 × 10 cm) on white paper. Stride length and step width were calculated.

### Statistics

Data are expressed as the means ± SEM. Statistical analysis was conducted using the software package SAS 8.1 (SAS Institute Japan Ltd., Tokyo, Japan). Statistical significance was determined by analysis of variance (ANOVA) followed by the Dunnett-type multiple comparison test, and p values of less than 0.05 were considered to be significant in the water maze task. Behavioural data in the rotating rod test were analysed by one- or two-way ANOVA with repeated measures, and p values of less than 0.05 were considered to be significant.

### Histopathology

Sections of tissues including the brain, spinal cord, heart, lung, liver, kidney, spleen and testis from mice (+/+, +/- and -/-; n = 7, 7 and 7, respectively) were fixed in 10% buffered formalin and embedded in paraffin. Each paraffin section of thick 4 μm was stained with hematoxylin and eosin (HE).

## Authors' contributions

KS is leading this project, and performed cDNA cloning, plasmid construction, antibody production, biochemical study, and wrote the manuscript. ET is also leading this project, performed the generation of knockout mice and behavioural analyses, and wrote the manuscript. TO and KY contributed to the generation of knockout mice. TN and JK supervised the project. All authors read and approved the manuscript.
